# Profiling CpG island field methylation in both morphologically normal and neoplastic human colonic mucosa

**DOI:** 10.1038/sj.bjc.6604432

**Published:** 2008-06-10

**Authors:** N J Belshaw, G O Elliott, R J Foxall, J R Dainty, N Pal, A Coupe, D Garg, D M Bradburn, J C Mathers, I T Johnson

**Affiliations:** 1 Institute of Food Research, Norwich Research Park, Colney, Norwich NR4 7UA, UK; 2 Newcastle University, Human Nutrition Research Centre, School of Clinical Medical Sciences, Framlington Place, Newcastle-on-tyne NE2 4HH, UK; 3 Wansbeck Hospital, Woodhorn Lane, Ashington, Northumberland, UK

**Keywords:** CpG island, DNA methylation, epigenetics, colorectal cancer, normal mucosa

## Abstract

Aberrant CpG island (CGI) methylation occurs early in colorectal neoplasia. Quantitative methylation-specific PCR profiling applied to biopsies was used to quantify low levels of CGI methylation of 18 genes in the morphologically normal colonic mucosa of neoplasia-free subjects, adenomatous polyp patients, cancer patients and their tumours. Multivariate statistical analyses distinguished tumour from mucosa with a sensitivity of 78.9% and a specificity of 100% (*P*=3 × 10^−7^). In morphologically normal mucosa, age-dependent CGI methylation was observed for *APC*, *AXIN2*, *DKK1*, *HPP1*, *N33*, *p16*, *SFRP1*, *SFRP2* and *SFRP4* genes, and significant differences in CGI methylation levels were detected between groups. Multinomial logistic regression models based on the CGI methylation profiles from normal mucosa correctly identified 78.9% of cancer patients and 87.9% of non-cancer (neoplasia-free+polyp) patients (*P*=4.93 × 10^−7^) using *APC*, *HPP1*, *p16*, *SFRP4*, *WIF1* and *ESR1* methylation as the most informative variables. Similarly, CGI methylation of *SFRP4*, *SFRP5* and *WIF1* correctly identified 61.5% of polyp patients and 78.9% of neoplasia-free subjects (*P*=0.0167). The apparently normal mucosal field of patients presenting with neoplasia has evidently undergone significant epigenetic modification. Methylation of the genes selected by the models may play a role in the earliest stages of the development of colorectal neoplasia.

Colorectal cancer is believed to develop primarily via the relatively well-characterised *adenoma–carcinoma* sequence (Lynch and Hoops, 2002). Although there is much variability in the overall profile of genetic abnormalities in any particular tumour (Jass, 2004), the transformation from normal mucosa to carcinoma is driven by the acquisition of mutations affecting genes involved in the control of cell proliferation, apoptosis and DNA repair. It is increasingly recognised that epigenetic changes, in which transcriptional silencing occurs independently of any change in DNA sequence, also play an important role. The most widely studied epigenetic event in colorectal carcinogenesis is the hypermethylation of CpG islands (CGI) associated with the promoter and first exon regions of tumour-suppressor genes (Esteller, 2003). A substantial minority of sporadic cancers have a very high level of CGI methylation affecting many genes; these are referred to as having the CGI methylator phenotype (CIMP), which appears to develop through a distinct pathway, having its origin in hyperplastic polyps rather than in adenomas (Issa, 2004). Hitherto, there has been a focus on the biology of the primary tumour and its immediate non-malignant precursor lesions rather than on the apparently normal epithelial cells in which the carcinogenic sequence begins. Nevertheless, there is a growing realisation that the emergence of focal lesions occurs in association with *field changes*, which can be defined as abnormalities of epithelial gene expression affecting the mucosal surface, rendering it vulnerable to neoplasia. This phenomenon of *field cancerisation* was first demonstrated in oral mucosa, in cases of multiple primary tumours or locally recurrent cancer (Slaughter *et al*, 1953), where it was believed to extend only a few centimetres beyond the tumour margin. However, more recent studies suggest that certain field abnormalities of the colon extend much further, perhaps encompassing the entire mucosal field (Polley *et al*, 2006).

Epigenetic modifications, in particular age-related CGI methylation, have been suggested to contribute to the field defect in the colon (Issa *et al*, 1994; Shen *et al*, 2005). Because aberrant CGI methylation occurs early, and is functionally linked with carcinogenesis, it offers the potential to provide biomarkers to assess an individual's risk of having or developing neoplasia. We have explored these issues using a quantitative methylation-specific PCR (QMSP) assay approach to measure the methylation status of 18 genes, previously demonstrated to play a role in colorectal carcinogenesis, and/or be affected by aberrant CGI methylation-induced gene silencing, in the morphologically normal mucosa of patients free from neoplasia at endoscopy, in adenomatous polyp patients and in both morphologically normal mucosa and tumour tissue from colorectal cancer patients. Using multivariate statistics applied to the often subtle differences in observed methylation status, we show that the apparently normal mucosa of cancer patients, and to a lesser extent of polyp patients, has undergone epigenetic modifications that distinguish them from patients free of neoplasia, and we identify a set of CGIs that contribute significantly to this field defect.

## Materials and methods

### Patients and biopsies

All volunteers were recruited from the gastroenterology outpatient and surgical lists of the Wansbeck General Hospital (Ashington, Northumberland, UK). Volunteers were either patients with previously diagnosed colorectal cancer or adenomatous polyps, or outpatients with no known gastrointestinal pathology who were undergoing flexible sigmoidoscopy or colonoscopy as a diagnostic procedure, typically for the investigation of nonspecific symptoms, such as abnormal bowel habit or unexplained rectal bleeding. Ethical approval for the project was received from the Northumberland Local Research Ethics Committee (Project reference NLREC2/2001) and consent was obtained in advance of the expected date of endoscopy or surgery. Experimental biopsies were collected from the sigmoid colon or rectum of the endoscopy patients and, for the cancer patients, samples of both normal mucosa (>10 cm from tumour margin) and tumour tissue were collected in operation theatre, immediately after surgery. All samples were immediately snap-frozen in liquid nitrogen and transferred to a −80°C freezer. Medical notes and pathology reports for each volunteer were reviewed 6–8 weeks after the procedure.

Samples of mucosa and tumour tissue were obtained from a total of 19 patients undergoing surgery for colorectal cancer (11 males and 8 females) with a median age of 74 years (range 52–86 years). Thirteen adenomatous polyp patients were recruited (10 males and 3 females) with a median age of 67 years (range 47–76 years). The ‘healthy’ group comprised 10 males and 10 females, median age 55 years (range 24–82 years), examined by flexible sigmoidoscopy (13 patients) or colonoscopy (7 patients), all of whom were diagnosed as being free from inflammatory bowel disease, hyperplastic polyps, adenomatous polyps and colorectal cancer.

### Bisulphite conversion of genomic DNA and analysis of CGI methylation

Biopsies were thawed, and genomic DNA was extracted and purified using a Genelute DNA extraction kit (Sigma-Aldrich, Gillingham, UK). Bisulphite conversion of DNA was carried out according to the method of Raizis *et al* (1995), which results in >99.8% conversion of unmethylated cytosines to uracil (Belshaw *et al*, 2004). CpG island methylation analysis was carried out on all the tissue samples for 18 genes, selected for their potential involvement in colorectal neoplasia and previously demonstrated to be affected by aberrant CGI methylation in colorectal cancer, using a QMSP assay developed in this laboratory. The selected genes were as follows: *SFRP1*, *SFRP2*, *SFRP4*, *SFRP5* (secreted frizzled-related proteins), *AXIN2* (Axis inhibition protein 2), *WIF1* (WNT inhibitory factor 1), *APC* (Adenomatous Polyposis Coli), *CDH1* (E-cadherin), *HPP1/TMEFF2* (Hyperplastic polyposis protein 1/Transmembrane protein with EGF-like and two follistatin-like domains 2), *ESR1* (Oestrogen receptor *α*), *MGMT* (*O*^6^-methylguanine-DNA methyltransferase), *MLH1* (*MutL* homologue), *p14*^*ARF*^, *p16*^*INK4a*^, *MYOD1* (Myogenic differentiation 1), *N33* (putative oligosaccharyl transferase), *DKK1* (Dickkopf-1) and *MINT31* (CGI 2 kb upstream of *CACNA1G*, a T-type calcium channel gene).

After bisulphite conversion, each CGI was amplified using primers not containing CpGs to give a pool of PCR fragments, each with a C or T at each CpG within the island. The proportion of PCR fragments containing C at each CpG, and therefore methylated in the genomic DNA, was determined using real-time PCR (MSP-M) with SYBR green as reporter, and primers containing Cs at the CpG sites, designed specifically to amplify the originally methylated alleles. By comparing the amplification threshold for the sample DNA with that obtained for a standard from a cloned PCR fragment derived from bisulphite-modified DNA that had been fully methylated by the treatment with *Sss*I methylase, the relative number of copies of methylated fragments was determined. The total number of PCR fragments present in the amplified pool was then determined from a second real-time PCR (MSP-T) with primers not containing CpGs, and the methylation status of the CGI was calculated. No-template, unmethylated and methylated controls were included in each assay.

Each CGI was first amplified by PCR in a reaction containing 50 ng bisulphite-modified DNA, 4 pmol of the appropriate primer (Supplementary Table S1), 10 *μ*l HotstarTaq master mix (Qiagen, Crawley, UK), 0.5 mM MgCl_2_ and water to 20 *μ*l. Following a 15-min hot start at 95°C, the PCR was performed using 35 cycles of denaturing at 95°C for 30 s, annealing for 1 min and extension at 72°C for 1 min. The T and M reactions were performed using 4 pmol of the appropriate primers (Supplementary Table S1), on 5 *μ*l of the amplified CGI fragment diluted 0.1–2.5 × 10^5^ times, in triplicate PCRs containing 10 *μ*l Immomix (Bioline, London, UK), BSA (1 mg ml^−1^), 0.5 mM MgCl_2_, 0.125 *μ*l 100 × SYBR green (Invitrogen, Paisley, UK), 0.4 *μ*l ROX reference dye (Invitrogen) and water to 20 *μ*l. Following a 7-min hot start at 95°C, PCRs were performed for 40 cycles of denaturing at 95°C for 30 s, annealing for 30 s and extension at 72°C for 30 s using an ABI 7300 machine (Applied Biosystems, Warrington, UK). A serial dilution (0.5–50 pg) of plasmid containing an insert amplified from fully methylated DNA was included on each plate as a standard. The level of allele-specific methylation was determined from standard curves, plotting *C*_t_ *vs* copy number for the T and M reactions for the serial-diluted plasmids. Percentage of methylation was calculated by dividing the number of methylated copies by the total number of copies. Samples were selected at random following T and M reactions and subjected to agarose gel electrophoresis to confirm PCR specificity and to check for primer–dimer formation.

### Preparation of plasmid standards for the QMSP assay

Placental DNA (Sigma-Aldrich) was artificially methylated with *Sss*I (CpG) methylase (New England Biolabs, Hitchin, UK) and *S*-adenosylmethionine according to the enzyme manufacturer's instructions. Following bisulphite modification, each CGI was PCR-amplified, using primers listed in Table S1 as described above. The amplified CGI fragments were gel-purified using a QIAquick gel extraction kit (Qiagen) according to the manufacturer's instructions, inserted into pCR4-TOPO TA cloning vector (Invitrogen) and used to transform *Escherichia coli* TOP10-competent cells (Invitrogen). Clones were re-suspended in 50 *μ*l water and screened using MSP-M PCRs as described above. Plasmid DNA was purified from positive clones using a QIAprep spin miniprep kit (Qiagen) and the inserts were sequenced using a BigDye terminator cycle sequencing kit and an ABI 3100 Avant sequencer (Applied Biosystems). Plasmids containing inserts with a C at each CpG were used as standards in the QMSP assays.

### Statistical analysis

Mean and median methylation levels for sample groups were compared by one-way ANOVA, followed by Tukey's test for the significance of differences. Where there was evidence of non-normality in the response variable, it was transformed to fit a normal distribution.

To search for differences in patterns of CGI methylation among the patient groups, we used binomial or multinomial logistic regression models fitted by regressing the tissue sample types on a number of genes as well as on age and sex. These variables were then ‘pruned’ using both backwards elimination using ANOVA-type tests, and an automated stepwise procedure for optimising the Akaike Information Criterion (AIC). ‘Leave-one-out’ cross-validation was used to estimate the classification error rate. This was compared with the expected rate given by the proportional chance criterion using an exact binomial test (one-sided) to test the null hypothesis that the given success rate of classification was no better than chance. Statistical analyses were carried out using Minitab Release 14 (Minitab Inc., State College, PA, USA) or ‘R’ (R Core Development team, http://www.R-project.org).

## Results

### Validity and sensitivity of the QMSP assays

The validity of the QMSP assay for each of the 18 CGIs was confirmed using a dilution series of artificially methylated DNA in unmethylated DNA (Supplementary Figure S1). For each assay, a linear decrease in the measured level of CGI methylation with dilution of the methylated DNA with unmethylated DNA was observed, indicating that the assay provides accurate determination of CGI methylation over several orders of magnitude, and also demonstrating an absence of any bias of unmethylated over methylated alleles introduced by PCR amplification (Warnecke *et al*, 1997). To illustrate the reproducibility of the QMSP assay, 10 independent assays for the *MLH1* CGI were performed on bisulphite-modified DNA from the cell lines HT-29 and SW48. The mean (±s.e.m.) levels of methylation measured were 3.57±0.31 and 99.6±1.6%, respectively.

### CGI methylation in matched apparently normal and tumour tissue

A comparison of the median levels of methylation in DNA from tumour tissue and paired apparently normal mucosa for 18 genes is presented in Table 1. For 13 genes, the levels of methylation in the tumour tissue exceeded those of the flat mucosa in the same patients, and for 7 genes these differences were statistically significant (*P*<0.05). For only one gene (*AXIN2*), the level of methylation was significantly lower in tumour tissue compared with flat mucosa (*P*<0.001).

We used binomial logistic regression modelling to compare the patterns of CGI methylation in the matched apparently normal and tumour tissues. Use of backwards elimination selected CGI methylation of *SFRP1*, *SFRP2*, *MYOD* and *MINT31* as the most informative variables . The model correctly classified 34 out of 38 (89.5%) samples giving a sensitivity for tumour classification of 78.9% with a specificity of 100%. The ‘proportional chance criterion’ predicts correct classification of 50% of the samples by chance alone; therefore, an exact one-sided test of 34 out of 38 samples correctly classified resulted in a *P*-value of 3 × 10^−7^. Thus, excellent discrimination between tumour tissue and normal mucosa from the same subjects was achieved by multivariate analysis of their patterns of CGI methylation, with a very high level of statistical significance.

### CGI methylation in the normal mucosa from neoplasia-free, adenomatous polyp and cancer patients

We compared the levels of CGI methylation for all 18 genes in the morphologically normal mucosa of patients with no detectable neoplasia, in polyp patients and in the cancer patients (Table 2). Some degree of methylation was detectable in the mucosa of neoplasia-free patients for all the genes assayed, although the levels varied considerably for different genes, from approximately 0.1% for *DKK1* to approximately 20% in the case of *N33*. For *APC* and *p16*, we observed significantly higher levels of methylation in cancer patients compared with neoplasia-free patients, with intermediate levels in the adenoma patients after adjusting for age and gender. The level of methylation was significantly lower in normal-appearing cancer mucosa compared with neoplasia-free mucosa for *ESR1*, *MINT31* and *WIF1* (Table 2).

**Figure 1:**
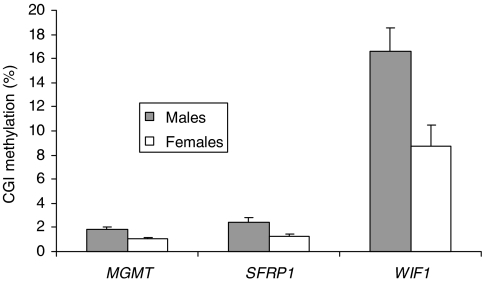
Differences in mean CGI methylation levels (±s.e.m.) in the apparently normal mucosa of males and females for *MGMT* (*P*=0.003), *SFRP1* (*P*=0.003) and *WIF1* (*P*=0.005). *P*-values were determined by ANOVA.

Regression analyses revealed statistically significant positive correlations between CGI methylation and age of all subjects for *APC* (*R*=0.331, *P*=0.015), *AXIN2* (*R*=0.334, *P*=0.014), *DKK1* (*R*=0.341, *P*=0.012), *HPP1* (*R*=0.484, *P*<0.001), *N33* (*R*=0.356, *P*=0.008), *p16* (*R*=0.355, *P*=0.008), *SFRP1* (*R*=0.532, *P*<0.001), *SFRP2* (*R*=0.345, *P*=0.011) and *SFRP4* (*R*=0.325, *P*=0.018). Stratifying the data by patient group led to a loss of statistical significance for some of the genes, but this is probably due to the small number of samples per group. Analysis of variance showed males were significantly (*P*<0.05) associated with higher methylation levels for *WIF1*, *SFRP1* and *MGMT* (Figure 1). Adjusting the data for age and patient group led to a loss of statistical significance for *MGMT*. The positive association of males with the methylation of *WIF1* and *SFRP1* remained statistically significant (*P*<0.01) but only in the neoplasia-free group.

We used multinomial modelling techniques to compare CGI methylation profiles in apparently normal mucosa samples from the cancer *vs* adenomatous polyp and *vs* neoplasia-free patients. The logistic regression model using automated backwards elimination with optimised AIC correctly classified 35 out of 52 (67.3%) of the samples with 16 out of 19 (84.2%, *P*=1.15 × 10^−5^) cancers, 5 out of 13 (38.5%, *P*=0.48) polyps and 14 out of 20 (70%, *P*=0.00125) neoplasia-free patients correctly identified. The variables selected as the most informative were CGI methylation of *HPP1*, *APC*, *SFRP4*, *p16*, *ESR1* and *WIF1*. Re-analysing the data with this model and grouping the neoplasia-free and polyp patients together as non-cancer subjects led to the correct classification of 29 out of 33 (87.9%, *P*=2.94 × 10^−10^) individuals. Thus, successful discrimination was achieved between the morphologically normal mucosa of cancer patients and those without cancer with a high level of statistical significance. However, this model was unable to distinguish the apparently normal mucosa of patients with adenomatous polyps from those with no neoplasia. Furthermore, logistic regression modelling for polyp patients *vs* neoplasia-free subjects correctly classified 8 out of 13 (61.5%) polyp patients and 15 out of 19 (78.9%) neoplasia-free subjects (*P*=0.0167). The variables selected as the most informative were CGI methylation of *SFRP4*, *SFRP5* and *WIF1.* Thus, discrimination between mucosal samples from neoplasia-free subjects and those with adenomatous polyps was relatively low, compared with the discrimination achieved between the morphologically normal mucosa of cancer patients and those without cancer, although still statistically significant.

## Discussion

A large number of studies have now identified a role for aberrant CGI methylation in gene silencing during colorectal carcinogenesis, with many CGIs reportedly affected in both tumours (Jones and Baylin, 2002) and in pre-cancerous lesions (Chan and Rashid, 2006). More recently, aberrant CGI methylation has been investigated in the normal mucosa of individuals with and without neoplastic lesions. In a comparison of patients with and without adenomas, the methylation of *p16*, *MLH1* and *MGMT* could not predict the presence of adenoma (Ye *et al*, 2006); in colorectal cancer patients, significant methylation was observed at the surgical margin (⩽10 cm from tumour) and was shown to be influenced by gender and by a polymorphism in the *DNMT3B* gene (Shen *et al*, 2005; Kawakami *et al*, 2006; Zhang *et al*, 2006; Iacopetta *et al*, 2007). Other studies have examined hyperplastic polyposis, where extensive methylation may underlie the condition (Minoo *et al*, 2006), and neoplasia-free, adenoma, hyperplastic polyp and cancer patients, where *ESR1* and *MLH1* methylation were generally higher in subjects with neoplasia, and were negatively associated with vitamin B-12 status (Al-Ghnaniem *et al*, 2007). In the present study, we have extended the investigation of methylation events in the mucosal field by using a QMSP assay to measure CGI methylation in 18 genes selected from the literature for their involvement in colorectal neoplasia, and shown previously to be affected by aberrant CGI methylation. Our findings suggest that changes in the methylation status of certain CGIs may contribute to, and therefore be used to define, the field defect associated with the development of colorectal neoplasia.

The QMSP method used here enabled us to measure accurately the often low levels of methylation for each CGI as a continuous variable. Using these data, we then applied multivariate statistical analyses to test the hypothesis that CGI methylation patterns in the morphologically normal mucosa of both cancer and adenomatous polyp patients differed significantly from those of patients with no neoplasia. The highly significant differences observed clearly demonstrate that the apparently normal mucosa of patients presenting with tumours or polyps has undergone epigenetic changes associated with the emergence of focal lesions elsewhere in the mucosal field. It should be noted that not all neoplasia-free patients underwent a full colonoscopy. However, 13 patients examined by flexible sigmoidoscopy were regarded as being at low risk of proximal lesions, and were discharged by the clinicians as free of neoplasia and inflammatory bowel disease. Although there is still a small risk that some patients harboured undiagnosed lesions in the proximal colon, this would presumably have tended to reduce the detected differences between groups.

The low levels of CGI methylation in morphologically normal mucosa have previously been dismissed as biologically insignificant on the grounds that they would have no measurable effect on gene expression (Ogino *et al*, 2006a, 2006b). However, QMSP measures CGI methylation, which probably occurs in an allele-specific manner (Supplementary Figure S2; Fackler *et al*, 2004; Belshaw *et al*, 2005). Because each colonic crypt is populated by clonal expansion of stem cells, aberrant CGI methylation is more likely to occur discretely in individual crypts. Furthermore, because crypts divide by fission, this mechanism might be expected to give rise to localised ‘islands’ or patches of hypermethylation within an otherwise normal (unmethylated) field (Preston *et al*, 2003). Thus, low levels of CGI methylation detected in mucosal biopsies may be of great biological significance, as they represent the mean of localised methylation events distributed unevenly within the epithelium. Recent evidence of *MLH1* CGI methylation affecting small patches of crypts in the unaffected mucosa of colorectal carcinoma patients provides support for this hypothesis (Nuovo *et al*, 2006).

Issa *et al* (1994) were the first to report that *ESR1* undergoes age-dependent methylation in the healthy human colon, and to propose that this form of epigenetic field effect may contribute to the increasing risk of colorectal cancer with age. In a subsequent work (Toyota *et al*, 1999), this group proposed a distinction between genes that commonly undergo age-dependent methylation (Type A) and those that become methylated only during the development of cancer (Type C). The panel of 18 genes used in the present study includes examples of both Type C and Type A genes as described by Issa and colleagues (Toyota *et al*, 1999). However, it should be noted that in a recent study, Kawakami *et al* (2006) observed age-related increases in CGI methylation for some genes classified as Type C by Toyota *et al*, which argues against any such rigid classification. In the present study, we observed statistically significant age-related increases in CGI methylation for *HPP1*, *p16*, *APC*, *AXIN2*, *SFRP1*, *SFRP2*, *SFRP4*, *N33* and *DKK1*, but contrary to previous studies (Issa *et al*, 1994; Kawakami *et al*, 2006; Al-Ghnaniem *et al*, 2007), the CGI methylation of *ESR1*, *MYOD* and *MLH1* showed no correlation with age in our patients. These findings may be due to analytical differences resulting in different CpGs within each island analysed or may indicate that the rates and sites of CGI methylation differ between populations, perhaps because of genotypic variation (Kawakami *et al*, 2006) or differing exposure to environmental factors, such as diet (Al-Ghnaniem *et al*, 2007) or commensal microorganisms.

Although CGI methylation of several genes was positively associated with age, age was not selected as a distinguishing variable by the logistic regression models. Furthermore, seven genes (*MLH1*, *DKK1*, *AXIN2*, *MGMT*, *N33*, *p14* and *CDH1*) were not selected by any of the models as features distinguishing between the disease states, indicating that their extent of methylation made no systematic contribution to the proposed ‘field defect’ in this study.

Higher methylation levels in the normal mucosa were associated with the male gender for *WIF1*, *SFRP1* and *MGMT*, which confirms the nonsignificant indication for male-associated methylation of *MGMT* observed by Shen *et al* (2005), but contradicts a recent report that the female gender was generally associated with higher CGI methylation levels in the normal mucosa of cancer patients (Kawakami *et al*, 2006). Interestingly, stratifying the data by patient group and adjusting for age showed that the male-associated increased methylation of *WIF1* and *SFRP1* was specific for the neoplasia-free subjects only.

The genes selected by the logistic regression models, used here as the most informative for distinguishing between the disease states, seem more likely to be mechanistically important for carcinogenesis. The genes distinguishing tumours from matched normal mucosa were *SFRP1*, *SFRP2*, *MYOD* and *MINT31*, whereas those distinguishing the morphologically normal mucosa of cancer patients from that of non-cancer (neoplasia-free+polyp) patients were *HPP1*, *APC*, *p16*, *SFRP4*, *ESR1* and *WIF1*, although *SFRP4*, *SFRP5* and *WIF1* could distinguish the normal mucosa of polyp patients from those without neoplasia. The observed CGI methylation of *HPP1* in this context is potentially of great interest. The methylation of *HPP1* was identified originally in hyperplastic polyps (hence the name), but it was also detected in colonic cancers, adenomas and in the normal mucosa, particularly of individuals bearing cancers with microsatellite instability (Young *et al*, 2001). *HPP1* methylation has also been detected in the dysplastic mucosa of ulcerative colitis patients (Sato *et al*, 2002). More recently, *HPP1* methylation was shown to occur early in Barrett's-associated neoplastic progression to oesophageal adenocarcinoma, and was predictive of progression risk (Schulmann *et al*, 2005).

There is good evidence that the aberrant activation of the Wnt signalling pathway is causal for approximately 90% of colorectal cancer (Giles *et al*, 2003). Previous studies (Suzuki *et al*, 2004) have suggested that CGI methylation-induced silencing of Wnt pathway antagonists may constitute an ‘epigenetic gatekeeper’, leading to constitutive activation of the Wnt pathway, and perhaps to ‘addiction’ in specific epithelial cells to its overactivity (Baylin and Ohm, 2006). This may favour the acquisition of mutations in downstream factors, eventually facilitating tumour progression. In the present study, the identification of CGI methylation of the Wnt signalling pathway regulatory genes *APC*, *SFRP1*, *SFRP2*, *SFRP4*, *SFRP5* and *WIF1* as significant features contributing to the discrimination of tumours from normal mucosa, cancer mucosa from non-cancer mucosa and polyp mucosa from neoplasia-free mucosa is consistent with this hypothesis. Coupled with the significant age-dependence of CGI methylation for six out of the eight Wnt antagonist genes studied here, these observations highlight the potential importance of these methylation events in the formation of the field defect.

CpG island methylation of both *WIF1* and *ESR1* genes made a significant contribution to the distinction between the normal mucosa of cancer patients and that of neoplasia-free patients. However, the average methylation levels were significantly higher in neoplasia-free subjects compared with cancer patients (Table 2) This observation suggests that epigenetic field changes may include both loss and gain of methylation. Our data for *ESR1* contradict those of Al-Ghnaniem *et al* (2007) who reported that *ESR1* was 19% more methylated in the normal mucosa of cancer patients than in those free of disease. The role of *WIF1* in the Wnt signalling pathway is poorly understood. *WIF1* binds to secreted Wnt ligands (Hsieh *et al*, 1999) and is frequently downregulated by CGI methylation in gastrointestinal cancers, including colorectal adenomas (Taniguchi *et al*, 2005). Recently, *WIF1* expression was observed throughout normal colonic crypts with enhanced expression in the stem cell zone at the crypt base (Byun *et al*, 2005). However, another study found that the *WIF1* expression was unique to adenomas (Gregorieff *et al*, 2005). Furthermore, the *WIF1* expression was elevated in intestinal adenomas compared with the normal epithelia of *Apc*^*Min/+*^ mice, and was observed in two human colon adenocarcinoma cell lines (Cebrat *et al*, 2004). This led the authors to propose a role for *WIF1* in facilitating adenoma growth, perhaps by inhibiting the generation and/or maintenance of the normal epithelial stem cell compartment.

As expected, the majority (13 out of 18) of CGIs were more methylated in tumours than in the matched apparently normal mucosa, with the difference being statistically significant for seven CGIs. Interestingly, on average, the *AXIN2* CGI was significantly less methylated in tumours compared with the matched apparently normal mucosa, and in only one tumour, it was more methylated than in matched apparently normal mucosa from the same person (6.8 *vs* 3.5%). *AXIN2* methylation has been reported by others to be correlated with microsatellite instability (Koinuma *et al*, 2006). Microsatellite instability is strongly associated with CIMP^+^ status (Weisenberger *et al*, 2006), and CIMP^+^ tumours have been reported to be associated with high CGI methylation levels in the normal mucosa (Kawakami *et al*, 2006). The analysis of mean *Z*-scores for the CGIs used previously to define CIMP status (*MLH1*, *MGMT*, *MINT31*, *p14* and *p16*; Minoo *et al*, 2006) suggests that only one of our tumours was CIMP^+^ (Supplementary Figure S3). Interestingly, this tumour also had the highest mean *Z*-score for all CGIs, indicating extensive methylation for many of the genes. However, the CGI methylation profile from the normal mucosa from this individual was not significantly different from those of the others (data not shown). In future studies, we intend to determine the performance of our statistical models with a larger set of cancer patients, in which CIMP status is defined.

In conclusion, the application of quantitative CGI methylation profiling, together with multinomial logistic regression modelling, demonstrates a role for CGI methylation as a feature of a generalised colorectal mucosal field defect associated with the presence of a neoplastic lesion. Such methylation events may cause ‘addiction’ of cells to aberrant gene expression and play a causal role at an early stage in the development of neoplasia (Baylin and Ohm, 2006). The application of this approach to a much larger population would almost certainly lead to more refined statistical models, and would enable the study of environmental factors, and their interaction with genotype, in the modification of DNA methylation patterns. Quantitative CGI methylation profiling for the identification and classification of field defects may also provide an objective approach to the early detection, or the assessment of risk, of colorectal cancer.

## Figures and Tables

**Table 1:** CGI methylation in 18 genes associated with colorectal carcinogenesis in the normal mucosa and tumours of cancer patients

	**Normal mucosa^a^**	**Tumour**	
	**CGI methylation (%) Median (range)**	**CGI methylation (%) Median (range)**	***P*-value^b^**
*APC*	1.24 (0.2–4.8)	2.59 (0.1–43.5)	0.137
*AXIN2*	2.68 (1.3–5.8)	0.91 (0.3–6.7)	4.72 × 10^−6^
*CDH1*	3.96 (0.3–9.9)	3.93 (0.2–14.2)	0.904
*DKK1*	0.21 (0–1.8)	0.21 (0–14.5)	0.637
*ESR1*	8.64 (4.6–11.5)	13.31 (5.5–28.9)	0.0019
*HPP1*	1.00 (0.3–2.9)	16.22 (0–43.4)	1.18 × 10^−11^
*MGMT*	1.21 (0.2–4.2)	2.46 (0.1–11.3)	0.302
*MINT31*	0.60 (0–4.3)	0.89 (0.2–19.5)	0.259
*MLH1*	1.01 (0.5–1.3)	0.99 (0.3–16.0)	0.232
*MYOD*	9.33 (2.5–31.0)	26.17 (4.5–58.7)	0.0002
*N33*	17.63 (4.0–44.0)	37.89 (11.3–57.8)	0.0040
*p14*	9.43 (1.6–18.1)	10.92 (0.2–84.0)	0.143
*p16*	0.33 (0–1.2)	0.52 (0–10.5)	0.284
*SFRP1*	1.90 (0.5–5.3)	8.86 (2.0–25.6)	1.61 × 10^−6^
*SFRP2*	2.11 (0.3–8.5)	15.49 (0.4–54.8)	9.74 × 10^−10^
*SFRP4*	0.96 (0–9.3)	0.94 (0–49.4)	0.987
*SFRP5*	0.50 (0–0.9)	0.70 (0–46.9)	0.0324
*WIF1*	1.75 (0.2–23.7)	3.81 (0.3–60.9)	0.300

CGI=CpG island.

a Values are median levels of methylation and ranges for 19 cancer patients.

b *P*-values are for the comparison of mean methylation levels from the mucosa with those from the tumour using ANOVA following transformation of the response variable where appropriate.

**Table 2:** CGI methylation in 18 genes associated with colorectal carcinogenesis in the normal mucosa of neoplasia-free, polyp and cancer patients

	**Neoplasia-free^a^ (*n*=20)**	**Polyp (*n*=13)**	**Cancer (*n*=19)**	
	**CGI methylation (%) Median (range)**	**CGI methylation (%) Median (range)**	**CGI methylation (%) Median (range)**	***P*-value^b^**
*APC*	0.40 (0.1–1.4)	0.55 (0–1.4)	1.24 (0.2–4.8)	0.013
*AXIN2*	2.10 (1.1–3.6)	1.95 (0.6–3.4)	2.68 (1.3–5.8)	0.556
*CDH1*	3.64 (0.2–9.6)	5.41 (1.3–8.5)	3.96 (0.3–9.9)	0.789
*DKK1*	0.14 (0–0.9)	0.17 (0–0.7)	0.21 (0–1.8)	0.070
*ESR1*	10.55 (3.8–16.5)	9.21 (4.4–14.0)	8.64 (4.6–11.5)	0.019
*HPP1*	0.50 (0–1.7)	0.96 (0–3.1)	1.00 (0.3–2.9)	0.708
*MGMT*	0.96 (0.3–4.0)	1.59 (0.6–4.8)	1.21 (0.2–4.2)	0.630
*MINT31*	2.20 (0.2–42.2)	2.22 (0.7–14.8)	0.60 (0–4.3)	0.016
*MLH1*	0.83 (0.1–2.9)	0.54 (0.2–5.3)	1.01 (0.5–1.3)	0.787
*MYOD*	11.43 (2.0–92.0)	21.00 (4.2–45.0)	9.33 (2.5–31.0)	0.301
*N33*	15.6 (5.3–38.4)	15.0 (8.5–27.6)	17.63 (4.0–44.0)	0.671
*p14*	6.07 (0–24.5)	6.37 (2.7–17.7)	9.43 (1.6–18.1)	0.622
*p16*	0.21 (0–0.6)	0.20 (0.1–0.6)	0.33 (0–1.2)	0.024
*SFRP1*	0.89 (0.1–4.1)	1.73 (0.3–3.7)	1.90 (0.5–5.3)	0.663
*SFRP2*	1.91 (0.2–6.6)	2.62 (1.4–8.5)	2.11 (0.3–8.5)	0.167
*SFRP4*	0.77 (0.2–3.9)	1.99 (0.5–4.6)	0.96 (0–9.3)	0.511
*SFRP5*	0.37 (0–2.0)	0.20 (0–1.5)	0.50 (0–0.9)	0.107
*WIF1*	11.76 (1.7–33.2)	19.18 (11.4–48.8)	1.75 (0.2–23.7)	0.005

CGI=CpG island.

a Values are median levels of methylation and ranges for mucosa samples from 20 patients free of neoplasia, 13 adenomatous polyp patients and 19 cancer patients.

b *P*-values are for the comparison of mean methylation levels from the mucosa of neoplasia-free patients with those from cancer patients using ANOVA. The analysis was performed after adjusting for age and sex, and following transformation of the response variable where appropriate.

## Supplementary Material

Supplementary FiguresS1–S3
