# Supplementary material for: Profiling CpG island field methylation in both morphologically normal and neoplastic human colonic mucosa
Source: Br J Cancer. 2008 Jun 10;99(1):136–42. doi: 10.1038/sj.bjc.6604432 (PMC2453007; doi:10.1038/sj.bjc.6604432)
Supplement: Supplementary FiguresS1–S3 [file 6604432x1.doc]

**Supplementary Figure S1.**

**(A) (B)**

**(C) (D)**

**(E) (F)**

**(G) (H)**

**(I) (J)**

**(K) (L)**

**(M) (N)**

**(O) (P)**

**(Q) (R)**

**Supplementary Figure S2.**

**(A)**

**(B)**

**(C)**

**(D)**

**Supplementary Figure S3.**
