# Supplementary material for: Profiling CpG island field methylation in both morphologically normal and neoplastic human colonic mucosa
Source: Br J Cancer. 2008 Jun 10;99(1):136–42. doi: 10.1038/sj.bjc.6604432 (PMC2453007; doi:10.1038/sj.bjc.6604432)
Supplement: Supplementary Table S1 [file 6604432x2.doc]

**Supplementary Table S1.** Primer sequences and annealing temperatures for the initial amplification of each CGI from bisulphite-modified DNA and for each QMSP T and M reaction.

| Gene | PCR | Forward (5’ – 3’) | Reverse (5’ – 3’) | Position relative to tsp (bp) | Annealing T (^o^C) |
| --- | --- | --- | --- | --- | --- |
| *APC*  *ESR1*  *HPP1*  *p16*  *MLH1*  *SFRP2*  *MYOD1*  *p14*  *CDH1*  *N33*  *MINT31*  *AXIN2*  *DKK1*  *WIF1*  *SFRP1*  *SFRP4*  *SFRP5*  *MGMT* | Initial  T  M  Initial  T  M  Initial  T  M  Initial  T  M  Initial  T  M  Initial  T  M  Initial  T  M  Initial  T  M  Initial  T  M  Initial  T  M  Initial  T  M  Initial  T  M  Initial  T  M  Initial  T  M  Initial  T  M  Initial  T  M  Initial  T  M  Initial  T  M | GTTAGGGTTAGGTAGGTTGT  GGGTGTTATTGGAGATAGAAT  TATTGCGGAGTGCGGGTC  GGGATGGTTTTATTGTATTAGATTTAAGGG  GTAGTTTAAGATTTTTTTGGAG  GCGAGGTGTATTTGGATAGTAGTAAGTTCGTC  AGAGTTTTTTTTTTATGGTAGTAGTT  AGAGTTTTTTTTTTATGGTAGTAGTT  GTTTTTCGCGTTTTCGGCGT  GGTTTTTTTTAGAGGATTTGAGGGATA  GGTTTTTTTTAGAGGATTTGAGGGATA  TTATTAGAGGGTGGGGCGGATCGC  TTAGATTATTTTAGTAGAGGTATATAAG  GAGAAATTTGATTGGTATTCAAGTTGTTTAA  TTAATAGGAAGAGCGGATAGC  GGTTAAGAAAATTTTGGTTGTG  GATGTTGTAGGGTTTGGTT  CGCGTTGTTTTTTCGGTGT  TAGGGGTATAGAGGAGTATTG  GGGTTGGTTGGTTAGTTT  CGTTTTTCGCGGATATAG  GTTTTAGTTTGTAGTTAAGG  TTATTTTTGGTGTTAAAGG  GTGTTAAAGGGCGGCGTAGC  GTGTAGGTTTTATAATTTATTTAGATT  GGTAGGTGAATTTTTAGTTA  TTAGGTTAGAGGGTTATCGCGT  TGGGTTTAGTAGTAGGATGG  GTGGAGGAGATATTGTTTGT  TACGTCGCGTTTTCGAAG  GAAAGGGTAATTAGGGAGA  GAAAGGGTAATTAGGGAGA  GCGGGAATTGAGACGATT  TTAGGATTTTTTGAATTTTTAAGGAGT  TTAGGATTTTTTGAATTTTTAAGGAGT  GTAGGGTCGAGTGGGGAAATATATC  TTTAGAAGGATTTAAGAGGGAGAAAG  GTTTTTTGGGAGGGAGATAA  GGGTTTTTACGAAATCGTGTC  GAGTGATGTTTTAGGGGTTT  GTATTGTGAATGTAGTTT  GGCGTTTTATTGGGCGTATC  GTTTTTTAAGGGGTGTTGAGT  GGGAGTTGATTGGTTG  CGGTCGTAGGAGTTTCGC  GGTTGTAGTTGTTAAGGGAG  GGGATAAATAGGGTTT  CGTAATGGTCGTGGTTGGTTGC  GTTTAGGTGGGTGGTAGTTTG  TTTGGTTTTAGTTTTAA  CGGTCGTTTGGTTTTAGTTTTAAGGC  GGTTTGGGGGTTTTTGATTAG  GGTATTAGGAGGGGAGAGATT  CGTAGTCGTTTCGAGTAGGATC | CCATAATAACTCCAACACCTA  CCATAATAACTCCAACACCTA  TCGACGAACTCCCGACGA  CTATTAAATAAAAAAAAACCCCCCAAAC  AACTTACTACTATCCAAATACACCTC  GTAAAAAAAACCGATCTAACCGTAAACCTACG  ACTCCCACAACACCATAACTA  AACATCCAAAAACTAAACTCAA  ATCATCCCGCGAACGACGA  CTACAAACCCTCTACCCACCTAA  CCAACCAACCCCTCCTCTTT  GACCCCCGAACCGCGACCGTAA  ATACCTTCAACCAATCACCTCAATA  ATACCTTCAACCAATCACCTCAATA  CTATAAATTACTAAATCTCTTCG  ACCTACTCCAACACCTCCTTC  AACCCAACAAATTAA ACA ACC  CGCTCTCTTCGCTAAATACGACT  TTCCTCACCCCTAACTTCT  TTCCTCACCCCTAACTTCT  ACGACCGACAACCCTA  TCCTCAATAACATCAACA  TCCTCAATAACATCAACA  AAACCCTCACTCGCGACGA  CAAACTCACAAATACTTTACAA  CAAACTCACAAATACTTTACAA  TAACTAAAAATTCACCTACCGAC  AAAAATCCATTCTACCTCCT  TTTTTCTTCTATCCTCCCC  TACGCGCCCAACTCCTA  AACACTTCCCCAACATCTA  AAATCAATCAATTCCCTCT  ACGCTTACGCCACTACGA  AATATAAAACACAACCTTCCAAAAAC  CTAAACTATATACAAATAAAACCAATTTC  GATACCATCAACTCCAAAAAAACG  AAATAAAAAATATCAAAAACCCCC  AAATAAAAAATATCAAAAACCCCC  AAAAAACCGAAATACTCCGAA  AACCTAAATACCAAAAAACCTAC  TAAAACTAACAAAACTAAC  CTAACGAAACCAACAATCAACG  CAAACTTCCAAAAACCTCC  CAAACTTCCAAAAACCTCC  GACTCCCGAAAATACGACG  AAAAAATTTCTTCCCCCAAACT  TCTAACACACAAAACAC  GAACAACTCCAATCCCGAACTCCG  AAAAAACAAAAAACCCTAAAAAAAA  AAAAAACAAAAAACCCTAAAAAAAA  AAACGAAACGCTCGAAGA AAATACG  CCTTTTCCTATCACAAAAATAATCC  CCTTTTCCTATCACAAAAATAATCC  GTACCCGAATAATCCTAAAAACG | -207 to +60  -9 to +60  -163 to -66  +392 to +666  +448 to +507  +480 to +601  +119 to +409  +119 to +215  +142 to +315  +48 to +379  +130 to +379  +131 to +281  -316 to +13  -112 to +13  -222 to -117  -28 to +467  +240 to +436  +68 to +158  -361 to +29  -163 to +29  -308 to -218  -27 to +227  +11 to +227  +20 to +140  -213 to +34  -82 to +34  -177 to -63  -158 to +362  +185 to +343  -57 to +172  -2065 to -1736  -2065 to -1782  -1990 to -1805  -603 to -305  -603 to -434  -487 to -398  -299 to +15  -190 to +15  -196 to -42  -436 to -21  -349 to -153  -369 to -167  -182 to +231  -66 to +231  -29 to +70  -123 to +128  -74 to +56  -58 to -2  -224 to +53  -56 to +53  -133 to -50  -565 to -368  -538 to -368  -501 to -417 | 59.5  59  65  58  58  66  56  58  67  62  63  74  52  60  56  58  62  63  59  58  60  56  52  67.5  58  55  64  55  58  60  58  60  62  54  60  63  56  60  64  54  42  64  59  56  63  50  52  70  56  43  63  60  58  61 |
