# Supplementary material for: Profiling CpG island field methylation in both morphologically normal and neoplastic human colonic mucosa
Source: Br J Cancer. 2008 Jun 10;99(1):136–42. doi: 10.1038/sj.bjc.6604432 (PMC2453007; doi:10.1038/sj.bjc.6604432)
Supplement: Supplementary Figure Legends [file 6604432x3.doc]

**Figure Legends**

**Supplementary Figure S1.** QMSP assay of CGI methylation in a dilution series of SssI-treated (fully methylated) DNA with untreated DNA for (A) *APC*, (B) *AXIN2*, (C) *CDH1*, (D) *DKK1*, (E) *ESR1*, (F) *HPP1*, (G) *MGMT*, (H) *MINT31*, (I) *MLH1*, (J) *MYOD*, (K) *N33*, (L) *p14* (M) *p16*, (N) *SFRP1*, (O) *SFRP2*, (P) *SFRP4*, (Q) *SFRP5* and (R) *WIF1*. The DNA was either placental or extracted from colonic tissue obtained from the tissue bank.

**Supplementary Figure S2.** Bisulphite genomic sequencing of plasmid inserts obtained from the cloning of PCR products obtained from the initial amplification of (A) *CDH1*(-213 to +34nt from tsp), (B) *SFRP1*(-183 to +231 from tsp), (C) *SFRP2* (-468 to +28nt from tsp) and (D) *WIF1* (-436 to -21nt from tsp) from bisulphite-modified DNA from 3 individuals who were free of neoplasia. The open circles refer to unmethylated CpGs and filled circles to methylated CpGs. QMSP-M primer annealing sites are represented by the arrows.

**Supplementary Figure S3.** Mean Z scores as determined by Konis*hi et* al, (2007) for all 18 CGIs and those used to define CIMP status (*MLH1, MGMT, MINT31, p14* and *p16*) in the 19 tumour samples.
